# Attitudes of Australian Patients Undergoing Treatment for Upper Gastrointestinal Cancers to Different Models of Nutrition Care Delivery: Qualitative Investigation

**DOI:** 10.2196/23979

**Published:** 2021-03-12

**Authors:** Kate Furness, Catherine Elizabeth Huggins, Helen Truby, Daniel Croagh, Terry Peter Haines

**Affiliations:** 1 Monash Health Nutrition and Dietetics, Monash Medical Centre Melbourne Australia; 2 School of Primary and Allied Health Care Faculty of Medicine, Nursing and Health Sciences Monash University Melbourne Australia; 3 Department of Physiotherapy School of Primary and Allied Health Care, Faculty of Medicine, Nursing and Health Sciences Monash University Melbourne Australia; 4 Department of Nutrition, Dietetics and Food School of Clinical Sciences, Faculty of Medicine, Nursing and Health Sciences Monash University Melbourne Australia; 5 School of Human Movement and Nutrition Sciences University of Queensland Brisbane Australia; 6 Upper Gastrointestinal and Hepatobiliary Surgery Monash Health Monash Medical Centre Melbourne Australia; 7 Department of Surgery School of Clinical Sciences, Faculty of Medicine, Nursing and Health Sciences Monash University Melbourne Australia

**Keywords:** qualitative, upper gastrointestinal, cancer, nutrition, mobile phone

## Abstract

**Background:**

Adults diagnosed with cancers of the stomach, esophagus, and pancreas are at high risk of malnutrition. In many hospital-based health care settings, there is a lack of systems in place to provide the early and intensive nutritional support that is required by these high-risk cancer patients. Our research team conducted a 3-arm parallel randomized controlled trial to test the provision of an early and intensive nutrition intervention to patients with upper gastrointestinal cancers using a synchronous telephone-based delivery approach versus an asynchronous mobile app–based approach delivered using an iPad compared with a control group to address this issue.

**Objective:**

This study aims to explore the overall acceptability of an early and intensive eHealth nutrition intervention delivered either via a synchronous telephone-based approach or an asynchronous mobile app–based approach.

**Methods:**

Patients who were newly diagnosed with upper gastrointestinal cancer and who consented to participate in a nutrition intervention were recruited. In-depth, semistructured qualitative interviews were conducted by telephone and transcribed verbatim. Data were analyzed using deductive thematic analysis using the Theoretical Framework of Acceptability in NVivo Pro 12 Plus.

**Results:**

A total of 20 participants were interviewed, 10 from each intervention group (synchronous or asynchronous delivery). Four major themes emerged from the qualitative synthesis: participants’ self-efficacy, low levels of burden, and intervention comprehension were required for intervention effectiveness and positive affect; participants sought a sense of support and security through relationship building and rapport with their dietitian; knowledge acquisition and learning-enabled empowerment through self-management; and convenience, flexibility, and bridging the gap to hard-to-reach individuals.

**Conclusions:**

Features of eHealth models of nutrition care delivered via telephone and mobile app can be acceptable to those undergoing treatment for upper gastrointestinal cancer. Convenience, knowledge acquisition, improved self-management, and support were key benefits for the participants. Future interventions should focus on home-based interventions delivered with simple, easy-to-use technology. Providing participants with a choice of intervention delivery mode (synchronous or asynchronous) and allowing them to make individual choices that align to their individual values and capabilities may support improved outcomes.

**Trial Registration:**

Australian and New Zealand Clinical Trial Registry (ACTRN) 12617000152325; https://tinyurl.com/p3kxd37b.

## Introduction

### Background

Adults diagnosed with cancers of the stomach, esophagus, and pancreas are at a high risk of malnutrition [1-9]. The nature of upper gastrointestinal (UGI) cancers directly affects digestive capacity and function due to tumor location and obstruction and inability to tolerate adequate volumes of oral intake confer risks of malnutrition, which are exacerbated by oncological, radiological, and surgical treatments [8,10]. Malnutrition increases the risk of complications. These include immune impairment leading to increased risk of infection, treatment toxicity resulting in dose reductions or cessation of chemotherapy, loss of muscle mass and decreased strength, increased complications associated with surgery, more hospital admissions, longer length of stay with decreased quality of life, and increased mortality [6,9,11,12].

In many hospital-based health care settings, there is a lack of systems in place to provide the early and intensive nutritional support that is required by these high-risk cancer patients. Our research team conducted a 3-arm parallel randomized controlled trial (RCT) to test the provision of an early and intensive nutrition intervention to patients with UGI cancers using a synchronous telephone-based delivery approach versus an asynchronous mobile app–based approach delivered using an iPad compared with a control group to address this issue [13].

A proposed definition of acceptability has been theorized by Sekhon [14] describing it as “a multi-faceted construct that reflects the extent to which people delivering or receiving a health care intervention consider it to be appropriate, based on anticipated or experienced cognitive and emotional responses to the intervention.” When we achieve acceptability with trials, we are more likely to see intervention adherence and engagement, and the proposed health outcomes of the intervention are more likely to be realized [15,16].

The majority of patients diagnosed with UGI cancers are aged over 60 years [17,18]. Given the increasing use of information and communication technologies (ICTs) such as mobile telephones, home computers, and the internet to provide health care, we need to be sure that older adults are not only willing but also able to use these technologies to receive their health care. Although these technologies are viewed as a bridge to the cost inefficiencies of in-person health care, allowing patients to access health care in their own homes, reducing inequities, and meeting their unmet health needs, they are potentially very costly interventions if patients do not accept and engage with the intervention being delivered [19]. A qualitative study delivered face-to face interviews with 123 people aged over 60 years examining the use of technology to evaluate their readiness to adopt health-related ICT found that increasing age negatively impacted ICT use, they needed ready access to support such as a spouse or family member, they were reluctant to adopt new technologies unless they were convinced of significant benefits, and their use of ICT was very basic [20]. Overall health was a moderating factor on ICT use, so the people who are most likely to benefit were the people least likely to use it [20].

Telehealth feasibility and acceptance have been examined by many reviews of telephone-delivered health-related interventions. Telehealth can be delivered via home phone or mobile telephone, so it has the potential to capture a larger audience of the population. Cancer-specific telehealth, looking at symptom management during head and neck cancer treatment, improves patients’ ability to self-manage their disease, including side effects from aggressive treatment [21]. It also provides patients with support and security [21]. A systematic review of 22 studies examining cancer survivors’ experience with telehealth health interventions found that it improved accessibility, that patients could raise concerns otherwise difficult to raise, and that it provided a safety net to receive support where required [22]. Other patients felt that it was time consuming, an additional burden, impersonal, and not sufficiently tailored to their individual needs and that lower engagement was found where the patient and health professional had not met in person before commencement of telehealth [22]. Another systematic review of telehealth interventions for cancer patients’ quality of life revealed a statistically significant improvement in quality of life and improved access to care, and telephone-based interventions were found to be superior to internet-based interventions [23]. COVID-19 has resulted in the rapid adoption of telehealth services to enable remote delivery of health care to comply with physical distancing laws and to keep vulnerable patients safe [24,25]. Thus, the impetus to understand the best way to deliver these services to enhance acceptability, enhance engagement, and improve patients’ health care outcomes has become even more urgent.

### Objectives

This study aims to explore the overall acceptability of an early and intensive nutrition intervention delivered via a synchronous telephone-based approach or an asynchronous mobile app–based approach.

## Methods

This qualitative study was set within a 3-arm RCT that examined two nutrition service delivery models, using a synchronous telephone-based approach or an asynchronous mobile app–based approach, to deliver a standardized early and intensive nutrition intervention to patients with UGI cancers (esophageal, gastric, and pancreatic) close to diagnosis, to determine improvements in quality of life, the protocol is available [13].

### RCT Interventions

The research dietitian contacted participants with their randomization information and set a date for the completion of their initial nutrition assessment. For some participants, this was carried out on the same day as the randomization call. The 18-week intervention commenced as soon as it was practicable after the participants’ diagnosis, consent, recruitment, and baseline data collection.

Participants received either intensive weekly or fortnightly collaborative and individually tailored nutrition interventions during their interaction with the dietitian. The synchronous telephone intervention was delivered using the participants’ home or mobile telephone. The mobile app intervention was delivered using a preexisting mobile app, MyPace. All participants were offered an iPad (Apple Air 2) device with internet connectivity prepaid from the study team that could be used to run the app; however, participants were also permitted to install the app on their own device, if they preferred to do so. MyPace allowed a messaging function for the participant and dietitian to communicate asynchronously and daily reminders to assist with self-monitoring of weight and completion of scheduled small steps (goals). Behavior change techniques used throughout the delivery of interventions in both groups were taken from the Behavior Change Technique Taxonomy v1 [26]. At the end of the 18-week intervention period, patients were able to access usual care.

### Design

Semistructured individual interviews were used to gather data from the perspective of participants in each of the intervention groups of the overarching RCT. The Standards for Reporting Qualitative Research were used in this study [27].

### Eligibility

Participants were eligible to participate if they were randomized into either of the intervention groups of the overarching RCT.

### Setting

The participants were drawn from 5 health services, including 3 tertiary public hospitals and 2 private hospitals in Victoria, Australia.

### Participants

Convenience sampling was used to select a group of participants with similar numbers involved in both the intervention arms of the overarching research trial.

### Procedures

One-on-one recorded semistructured interviews were conducted by a researcher (CH) via telephone. Interviews were conducted between September 2017 and June 2019. The interviews were designed to be completed within 30 minutes to ensure that they were not overly taxing to the participants.

### Method of Approach

At the conclusion of active interventions, a convenience sample of participants was asked if they would consent to participate in a postintervention interview with another researcher (CH). Of those who accepted, their data was entered into an intervention completion data file with their name, contact details, and intervention group details. The researcher (CH) contacted the participants by phone. Participants who did not respond to the first phone call were then contacted with another follow-up call, email, or text message. Participants who did not respond to the follow-up were not contacted again (17/37, 46%).

### Measurements

An interview guide (Table 1) was developed by the researcher (CH) after feedback from the overarching research trial investigator team. Interviews were conducted between September 2017 and June 2019. Immediately at the conclusion of each interview, the interviewer (CH) made reflective field notes.

**Table 1:** Semistructured postintervention interview questions.

Questions	Logic
As someone who has cancer, what is it like for you managing your nutrition?	Living with cancer
Tell me about the experience you had as a participant in this study. Did it meet your nutritional needs?	Relevance to the patient
What was it like for you being contacted by the dietitian frequently? Tell me what was challenging. Would you change anything (throw something out, add something in?) What did you like? (iPad group) What was it like learning a new App?	Self-management practice
Tell me what it was like communicating with a health profession using the phone (or iPad). What helped or hindered communication between you and the dietitian? What would have made this experience better for you? Describe any challenges you had communicating with the dietitian. What do you need to facilitate communication? What could we have improved the way we delivered the nutrition to you?	Communication
If you could design this service, what would be the key features of the service? Tell me about the scheduling of the consultations. How important is flexibility? What could we have done to support you better?	Unmet care needs
What motivated you to take part in this study?	Motivation
Is there anything else you’d like to tell me about that relates to your experience throughout the intervention?	Overall experience
What role did your family play in your nutrition care during the study period?	Social influences
Did you contact the dietitian as often as you wanted to? What motivated or stopped you from using the app?	Contact
Did you have any problems using the app or contacting the dietitian? If you had problems, what were they? How did you solve the problem?	Technical problems
Did any of your family members help you with the app or dietetic consultations? How important was it to you to be able to involve someone else in this service? Would they like to share with me their experience of the intervention?	Family/career engagement

### Data Preparation

The recordings of the interviews were transcribed verbatim and deidentified for analysis. Interview recordings and transcriptions were stored in a secure cloud-based repository.

### Researcher Positioning

The researcher KF is a senior clinical dietitian in general, UGI, and hepatobiliary surgery at one of the tertiary hospitals in this study. The delivery of nutrition intervention in the concurrent intervention study was also conducted by this researcher (KF).

### Reflexivity

As an insider in this research study, KF was deeply embedded with emotional investment in the provision of health care to the intervention population and a member of the health professional team [28]. KF continually evaluated her subjective and automatic responses and how they were intertwined with how she ultimately interpreted and actively constructed knowledge throughout the research process [29].

### Rigor

Standardized interview questions were developed before the commencement of interviews and refined throughout to promote rigor in data collection. A wide variety of participants across both intervention groups with different cancer types, ages, and genders increased the dependability of the data collection. Verbatim transcription of interview audio recordings and data analysis verification by a second author (CH) supported accuracy and reliability.

### Analysis

**Figure 1:**
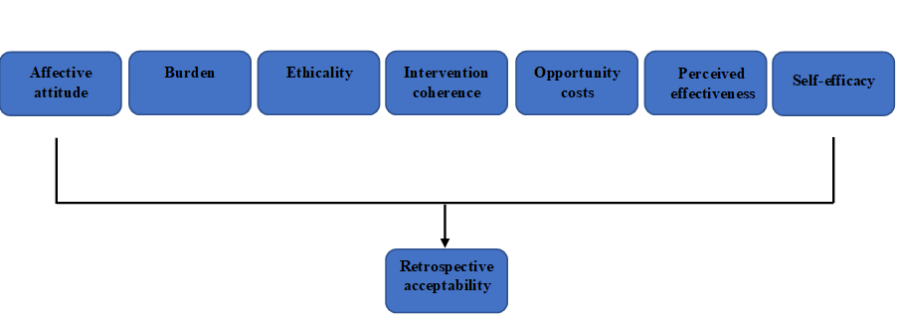
The Theoretical Framework of Acceptability. Data were collected retrospectively and explored using each of the 7 acceptability constructs.

The data analysis framework was based on the 7 constructs of the Theoretical Framework of Acceptability: affective attitude, burden, intervention coherence, ethicality, opportunity costs, perceived effectiveness, and self-efficacy (Figure 1) [15]. Hand coding of all interviews was used as the first pass of the deductive coding of framework constructs. Researchers (KF) and (CH) met to discuss the coding accuracy of the two interviews until a consensus was reached. NVivo Pro 12 Plus was used to sort and analyze the data. This process allowed coding to be reexamined carefully.

### Ethics Approval

Conduct of the study at all sites was approved and reviewed by the Monash Health Human Research Ethics Committee (HREC/16/MonH/290). Site-specific authorization has been granted for all sites (Monash Health, Jessie McPherson Private Hospital, Cabrini Health, Eastern Health, and Peninsula Health). Participants provided written informed consent to participate in the study.

### Consent to Publish

Participants provided written informed consent to participate in the study, which is the approved consent process by the main ethics committee (Monash Health Human Research Ethics) and the other sites (Cabrini Health, Eastern Health, and Peninsula Health).

### Data Availability

Where requested by publishers, deidentified participant data may be stored in a public repository. These data will be restricted and deidentified so that combinations of data cannot be used to potentially identify participants.

## Results

### Participant Sample

A total of 37 participants consented to be interviewed, and a total of 20 interviews were completed, providing a total response rate of 54%. There were 15 participants who were unable to be contacted via telephone despite consenting to be contacted for postintervention interviews; these participants were nonresponders. Two participants subsequently withdrew their initial consent to participate. All interviews were conducted via telephone.

Information power was used as a tool to assist with sample size determination, as described by Malterud [30], which indicates that lower numbers of participants are required when more relevant information is held by the sample.

Demographics of the interview sample are shown in Table 2.

**Table 2:** Characteristics of the interview sample.

Participant characteristics		Total participants (N=20), n (%)	Telephone (n=10)	Mobile app (n=10)
**Gender**				
	Female	7 (35)	3	3
	Male	12 (60)	7	6
	Other	1 (5)	0	1
**Age (years)**				
	50-59	5 (25)	3	2
	60-69	8 (40)	3	5
	70-79	7 (35)	4	3
**Cancer type**				
	Gastric	6 (30)	3	3
	Esophageal	5 (25)	3	2
	Pancreatic	9 (45)	4	5
**Education**				
	Year 1-12	8 (40)	4	4
	>Year 12	12 (60)	6	6
**Baseline Patient-Generated Subjective Global Assessment^a^** **score**				
	<9	9 (45)	5	4
	≥9	11(55)	5	6
Mortality		9 (45)	5	4

^a^Patient-Generated Subjective Global Assessment: scores ≥9 indicate a critical need for nutrition intervention and symptom management [31].

There were a total of 20 participants, 10 participants from each group (7 female, 12 male, and 1 other). Five participants were aged between 50 and 59 years, 8 were aged between 60 and 69 years, and 7 were aged between 70 and 79 years.

### Self-Efficacy, Low Levels of Burden, and Intervention Comprehension Required for Intervention Effectiveness and Positive Affect

Participants perceived the intervention as effective when particular conditions related to the construct of acceptability were met. They positively viewed the intervention when there were low levels of participant burden, when they understood how the intervention worked, when they felt that it aligned with their value system, and when they were confidently able to perform the tasks and behaviors required of them. If these conditions were not met, the intervention was perceived as having little benefit. This was described more commonly by the participants in the mobile app group. People felt that their age, skill level, and familiarity with technology affected their participation in the intervention. The introduction of new technology often did not correspond to their daily habits and normal behaviors. Therefore, it required them to learn a new skill and incorporate this into their lives, at the same time as receiving treatment for cancer. This was often too challenging for participants and was a significant barrier to engaging in eHealth interventions. Even when participants had high levels of technology skill, if the intervention delivered via the mobile app did not align with their value system of communication, they were less inclined to use it:

I’ve learnt so much from interacting with [Dietitian], and her suggestions to how I can improve my diet and what have you. I have learnt a lot from her, and I appreciate that, and I think that’s the best part of it, that a patient has a chance to interact with a professional like that, and be able to help themselves.

Participant 10, 61-year-old female

I’m at that age where although I understand computers and I can do just about anything with them, to me there’s nothing better than sitting down and having a face-to-face or a voice-to-voice conversation. So maybe it’s a bit of an age thing with me.

Participant 1, 70-year-old male

–Na, not bothering with an app, because I don’t even have anything on my phone. I’ve got app things there and stuff I’ve never even looked at them. All these things pop up and I’m like, “You know what? I don’t want to be on me phone.”

Participant 4, 71-year-old female

Many participants used a family member or spouse or partner to increase their engagement with the intervention on their behalf. This occurred in both the synchronous and asynchronous groups. The burden of the intervention was perceived to be higher when English was not their first language or when symptoms from the disease and/or treatment were high. This was often coupled with lack of self-efficacy and skills to use technology:

He didn’t do any of it. He is good on the computer, but he’s limited, he doesn’t sort of muck around as much as I do.

Participant 8, 73-year-old male

For the mobile app group, it was found that ease of use of hardware and operating systems was important for the acceptance of the mode of delivery. Participants who chose to use their own devices instead of the provided iPad had issues with service delivery, as the app was not supported by Android devices. Participants who experienced service disruption due to technical issues with the technology itself, including issues with loading the app on their home computers, tablets, and smartphones; logging in; and internet connectivity, became disengaged and frustrated.

There was a small subgroup of participants who did not realize that they were not participating in the intervention as intended, reflecting that they poorly understood the intervention. They perceived the intervention as low burden and believed they had high self-efficacy to complete what was required of them, resulting in affective attitude and perceived effectiveness. The intervention was not actually delivered to them as was prescribed because they did not fully understand the requirements for complete participation:

I think I was weighing myself twice a week.

Participant 9, 61-year-old female

The analysis using the Theoretical Framework of Acceptability constructs has been illustrated in Table 3.

**Table 3:** Analysis using the Theoretical Framework of Acceptability constructs.

Theoretical framework of acceptability: construct	Definition	Participants, n (%)	Code frequency: for	Defining quote	Code frequency: against	Defining quote
Affective attitude	How an individual feels about the intervention	18 (90)	122	“The dietitian was quite competent in answering everything overall over that 18 weeks and I think she just kind of set the groundwork for me and now I kind of know what I need to do to best look after myself.” (Participant 14, 78-year-old male)	12	“It was not important to have any external information about my food and my diet because I knew it myself.” (Participant 13, 71-year-old female)
Burden	The perceived amount of effort required to participate in the intervention	19 (95)	32	“I think really important because you don’t have to go anywhere. You don’t have to look respectable, when it’s going to an appointment for the chemo or for the doctors or oncologists et cetera. Just to have that informal phone call, is great.” (Participant 1, 70-year-old male)	31	“Well, I didn’t really go on the app at all. I’m not really a technical person.” (Participant 18, 72-year-old male)
Ethicality	The extent to which the intervention has a good fit with the individual’s value system	6 (30)	9	“Well, all I can say is that that was very positive. You know, I was more than happy with speaking to [Kate] via the telephone. I didn’t feel as though I needed to sit across the desk from anybody and speak face to face. No, I’m very happy with it, you know, that mode of communication.” (Participant 1, 70-year-old male)	2	“I’m at that age where although I understand computers and I can do just about anything with them, to me there’s nothing better than sitting down and having a face-to-face or a voice-to-voice conversation. So maybe it’s a bit of an age thing with me.” (Participant 7, 68-year-old female)
Intervention coherence	The extent to which the participant understands the intervention and how it works	13 (65)	17	“Primarily it’s support to patients with proper diet and maintaining weight and all that sort of thing.” (Participant 3, 75-year-old female)	6	“I didn’t access anything, I had you or somebody ring me up and we went through the questions verbally on the phone.” (Participant 10, 61-year-old female)
Opportunity costs	The extent to which benefits, profits, or costs must be given up to engage in the intervention	6 (30)	3	“And you know, I made myself available. Otherwise, I was always able to leave her a message. I had her number and was able to leave her a message and you know, we could reconvene at another time.” (Participant 2, 63-year-old male)	3	“I prefer the phone – because I don’t have the time.” (Participant 4, 71-year-old female)
Perceived effectiveness	The extent to which the intervention is perceived as likely to achieve its purpose	17 (85)	120	“Well, like I said, (the dietitian) would send me an email and suggest various things that I can do. And I emailed her and told her what I was doing, and she encouraged me to do those things, because they were important. So, it was a case of between the two of us, we bounced back on each other. And I got what I needed out of it.” (Participant 12, 70-year-old female)	19	“The small steps thing that I was supposed to be filling out, I couldn’t record it every day.” (Participant 16, 61-year-old male)
Self-efficacy	The participant’s confidence that they can perform the behavior(s) required to participate in the intervention	11 (55)	14	“No, it wasn’t a problem, because I’m very tech-savvy, I don’t have a problem with any computer or iPad.” (Participant 8, 73-year-old male)	13	“I’m 80 darling and I would stuff it up.” (Participant 5, 79-year-old male)

### A Sense of Support and Security Through Relationship Building and Rapport

Participants who engaged well with the intervention frequently reported feeling a sense of support and security and felt that someone was there if they needed them. This was built through the frequent nature of interactions with participants, which facilitated the formation of a trusting relationship. Participants eagerly awaited their appointments with the dietitian, and the cessation of the intervention at 18 weeks shocked and saddened them, as they felt a keen sense of loss. These psychological and emotional effects on participants at the conclusion of trials have been reported in a number of other qualitative articles examining postintervention transition to usual care, with participants reporting a sense of loss, disappointment, anxiety, and isolation [32,33]. This highlights the need to incorporate how to manage the psychological impacts of abrupt cessation of intensive interventions on trial participants in the future [33]. Despite this sense of loss, they also felt well prepared to manage their nutrition needs moving forward even if more treatment was planned (eg, additional rounds of chemotherapy). Participants often shared intimacies of their symptoms that they may have found embarrassing to discuss without the close rapport and nature of non-face-to-face interventions where some level of anonymity was maintained. The conduct of the dietitian delivering the intervention in both groups was remarked as important for building rapport. Participants valued what they referred to as high levels of empathy through nonjudgmental, kind, and encouraging communication. Furthermore, participants reported that the strong rapport enabled them to offload their concerns and reduce their loneliness without burdening those close to them:

When the dietitian used to ring, she would listen, she was never judgmental. If I was feeling rotten and I swore on the phone, she didn’t say anything. It’s a very lonely journey.

Participant 1, 70-year-old male

I think it’s benefited us, and him in particular, because he felt like he had someone watching over him, in respect of that, and it didn’t cost us anything, it didn’t take up too much time, and it was a great help. I felt like it was a guardian angel over him. So, to have that support, it was wonderful.

Participant 8, 73-year-old male

I really missed it. I really missed that conversation via the iPad because I could express myself quite well, I thought.

Participant 2, 63-year-old male

### Knowledge Acquisition and Learning Enabled Empowerment Through Self-Management

Participants reported developing knowledge on how to make appropriate decisions to self-manage their nutrition impact symptoms and often complex dietary modifications alongside their treatment. They felt this was through the provision of supportive, targeted educational contact that was easy for them to understand with guidance and repeated messaging. Many participants had a high symptom burden, including fatigue, anorexia, and diarrhea, many of which were considered very distressing, reducing their quality of life. Some participants reported that they initially thought that these were inevitable, that they needed to cope with them, and that they could not be remedied. Participants described that the detailed explanations of disease process and treatment effects on the body allayed their anxieties, whereas timely identification and intervention as a team allowed them to take more control of their symptom management:

And the body not doing what I was telling it. It was doing what it wanted to. And tough luck with what I was thinking, virtually. So that required understanding, information, and support.

Participant 4, 71-year-old female

The dietitian was quite competent in answering everything overall over that 18 weeks and I think she just kind of set the groundwork for me and now I kind of know what I need to do to best look after myself.

Participant 14, 78-year-old male

### Convenience, Flexibility, and Bridging the Gap of Hard-to-Reach Individuals

Given the physical and psychological burden of cancer treatment, participants overwhelmingly preferred the convenience, flexibility, and accessibility of a home-based intervention. Many people lived far away from tertiary hospitals where many of their face-to-face health provider interactions took place, which was a significant burden for these participants. They expressed the favorable conditions of not having to get dressed or have a formal appointment, and these synchronous or asynchronous interactions likely increased their level of comfort and relaxation with the intervention, particularly related to information sharing. Most participants did not allude to the financial ramifications of seeing health care providers, but one participant saw the benefit of being offered a free service. Participants in both groups enjoyed the flexibility of being able to schedule appointments that fit with their lives or to send messages via the app late at night when they were experiencing concerns or anxieties, knowing that it would be attended too quickly. Some participants thought that the 18-week intervention should have been delivered as 18 individual sessions when they required it, rather than over consecutive weeks. As they were in and out of hospital for treatment, they may have missed weeks, and they felt that 18 weeks did not often cover their treatment duration:

If I’m worried about something at 10 o’clock at night I can send an email off and the dietitian will get back to me the next day. That takes the anxiety out of that situation I think as much as anything, so it was good.

Participant 16, 61-year-old male

You know, some days it’s a struggle to go to the garage and jump in the car let alone, you know, go to – go sit in the car for half an hour or 45 min to get somewhere, it’s just hard to do, so to have that flexibility of being able to say, you know, well can we do it on such-and-such a date, yeah, makes a hell of a difference.

Participant 15, 52-year-old male

One participant described the requirement to escalate concerns as they arose via the telephone despite being randomized into the mobile app group. The store-and-forward nature of the mobile app group meant that concerns may not have been actioned in a period that was deemed fast enough to respond to urgent issues:

Yes. I suppose I felt like my message was being heard more urgently if I spoke to someone, rather than just did it via the app. I don’t know that I had that confidence that it was going to be picked up straight away. I suppose that’s just me, I shouldn’t have done that, but anyway.

Participant 1, 70-year-old male

## Discussion

### Principal Findings

Delivery of an early and intensive nutrition intervention via telephone or mobile app were both largely acceptable delivery modes for people with UGI cancers. However, it was apparent that specific requirements need to be met for this to be the case. Participants required a perception that their self-efficacy was high, that the intervention was of low burden, and that they understood what was required of them to feel that the intervention was acceptable. This led to participants having positive affect related to the study, and they viewed the intervention as effective in meeting their needs. Some participants did not find the mobile app delivery mode acceptable. They cited their age, perceived and actual skill level, technological savviness, and delivery mode requiring a significant shift from normal daily activities as major barriers to engagement. Adapting the intervention to improve perceived fit with the individual may include modification of technology and mobile app with extensive consumer engagement, allowing people to choose their own delivery mode to suit their communication preference and allowing participants to choose the timing and quantity of dietitian contact. Ascertaining whether participant autonomy in choosing the timing and delivery of the contact for both delivery approaches would impact the effectiveness of the nutrition intervention requires further examination.

To date, few studies have investigated the application of eHealth during the active treatment phase of cancer, with even fewer studies using it in the UGI cancer cohort [34]. The penetration of smartphones in Australia is at an all-time high, with 91% of the population having a smartphone, including 77% of people aged over 55 years [35]. This aligns well with the UGI cancer patient population, where most diagnoses are made in those aged over 60 years [17,36,37]. Having a smartphone does not necessarily correlate with the high-level use of the technology embedded within them. Older adults are the least likely age group to engage with technology, as they are often late adopters of new innovations [38,39]. This may be a transient concern as technology becomes increasingly embedded in our everyday lives.

Low burden through ease of use was highlighted as an important consideration for our participants, supported by evidence that individuals may cease use of technology if the benefits are not perceived early on in its use [38]. Participants reported issues with loading the app, logging on, and internet connectivity, all of which would have increased the burden of use for many participants unfamiliar with the use of technology. This, in turn, may have increased motivational barriers and decreased self-efficacy. Older people are less likely to ask for help, as they do not want to burden others despite technical assistance being offered by the research team [19]. Many of our study participants required the assistance of family members, such as their spouses, to undertake the communication element of the intervention on their behalf when they deemed engaging with the eHealth component too challenging for them individually; interestingly, this was predominantly female partners/carers. A study of older Dutch adults exploring teleconferencing intention and capability to use found that family support positively impacted frequency of use and self-efficacy through mastery [40]. Engaging family members more actively may be an important mechanism to enhance participants’ use of technology, and it needs to be considered in intervention delivery in the future.

Participants’ nutrition, symptom, and pharmacological management information needs were high, and they requested easy-to-understand, tailored information provided by a confident and supportive dietitian to enhance their self-management. This aligns with patients with cancer generally wanting to receive information that helps them [41]. These aspects of nutrition intervention delivery are essential to meet the acceptability constructs of affective attitude and perceived effectiveness. A survey of 185 patients with cancer exploring satisfaction with the information provided found that only 50% were happy with the level and amount of information they were given throughout their treatment journey [42]. Many of our study participants required ongoing, repeated messaging throughout the course of their 18-week treatment journey to actualize self-management. They found that both methods of delivery allowed them to communicate better with their dietitian, which enabled shared decision making.

Limitations of this study include the possibility of selection bias due to participant mortality before the opportunity to participate in this evaluative aspect of the overall study. Similarly, the absence of data from those who were uncontactable and those who withdrew their consent may also have introduced bias. Participants who were amenable to participating in the interviews may have been more likely to view the intervention positively. There was a risk that people provided obsequious responses in this study, as the same investigative team that developed and delivered the intervention conducted this evaluation. To minimize this potential risk, an investigator who was not involved in the delivery of the intervention with participants undertook the data collection interviews. Similarly, there was a risk that investigators involved in the analysis of our data may have sought to overplay positive feedback and underplay negative feedback, as they were involved in the delivery of the intervention. We verified the data analysis and coding by a second author who was not involved in the delivery of the intervention to mitigate this risk.

Future research needs to focus on evaluating the relationships between acceptability, engagement, and use to ensure that eHealth nutrition interventions are effective in their intended health outcomes, both long and short term. Similarly, evaluating the cost-effectiveness of these interventions will be critical to inform future uptake of novel eHealth service delivery models. Central service delivery models versus those delivered through individual health services need to be explored in detail.

### Conclusions

This study has shown that early and intensive eHealth nutrition models delivered via telephone and mobile app are acceptable to patients undergoing treatment for UGI cancers when the core requirements for individual fit are realized, including self-efficacy, low levels of burden, and comprehension of the intervention to bring about effectiveness and positive affect. Convenience, knowledge acquisition, improved self-management, and support were key benefits for participants to engage with eHealth. Future interventions of this nature should focus on home-based interventions as an adjunct to usual health care. Simple, easy-to-use technology with technical support, which allows individual choice with respect to the mode of delivery of the intervention so that it aligns with the individual’s intrinsic value system, will enhance acceptability.

## Abbreviations

ICT: information and communication technology
RCT: randomized controlled trial
UGI: upper gastrointestinal
